# Maxillary Labial Frenulum Morphology and Maxillary Anterior Dental Caries in Children: A Scoping Review

**DOI:** 10.3390/children13101340

**Published:** 2026-10-02

**Authors:** Mehmet Ali Tuna, Faezeh Naderloo, Ufuk Kesmez, Ayşegül Aydın Tanas, Sera Simsek Derelioglu

**Affiliations:** 1 Department of Periodontology, Faculty of Dentistry, Atatürk University, 25240 Erzurum, Türkiye; f.naderlo@yahoo.com (F.N.); ukesmez25@gmail.com (U.K.); aysegulaydin25@gmail.com (A.A.T.); 2 Department of Pediatric Dentistry, Faculty of Dentistry, Atatürk University, 25240 Erzurum, Türkiye

**Keywords:** maxillary labial frenulum, maxillary anterior dental caries, pediatric dentistry, frenulum attachment, early childhood caries, scoping review

## Abstract

**Highlights:**

**What are the main findings?**

- Evidence from six analytical cross-sectional studies was heterogeneous, with no consistent association between MLF attachment and maxillary anterior caries.
- Only one included study reported a multivariable analysis, and no MLF attachment category showed a statistically significant adjusted association with caries severity.

**What are the implications of the main findings?**

- MLF attachment should not be used as a stand-alone marker of maxillary anterior caries risk.
- Current evidence does not support recommending frenectomy solely for the purpose of preventing maxillary anterior caries.

**Abstract:**

**Background/Objectives:** The relationship between maxillary labial frenulum (MLF) morphology and caries of the adjacent maxillary anterior teeth remains uncertain. This scoping review mapped the available evidence in children. **Methods:** Following Joanna Briggs Institute (JBI) guidance and PRISMA-ScR, PubMed/MEDLINE, Scopus, Web of Science, and TR Dizin were searched up to August 2026, plus reference lists. Analytical studies in participants under 18 years of age were eligible. Two reviewers independently screened records and verified charted data; included studies were appraised post hoc using the JBI analytical cross-sectional checklist. **Results:** Of 68 unique records, six cross-sectional studies (1659 participants aged 6 months–16 years) were included. Five used the Placek classification or an equivalent and one the Kotlow scale; caries outcomes ranged from approximal lesions to ICDAS-based severity. Three studies reported bivariate differences without a consistent gradient, whereas three found no significant association. In the only adjusted analysis, no attachment category differed significantly from the mucosal reference; the others were unadjusted. **Conclusions:** Evidence is limited and heterogeneous. MLF attachment should not be used as a stand-alone caries risk marker. Current evidence does not support recommending frenectomy solely for the purpose of preventing maxillary anterior caries; this reflects insufficient evidence of benefit rather than evidence of ineffectiveness. Prospective studies with standardized anatomical and functional assessments are needed.

## 1. Introduction

The maxillary labial frenulum (MLF) is a fold of mucous membrane and underlying connective tissue extending from the inner aspect of the upper lip to the anterior maxillary alveolar mucosa and gingiva [1]. Its level of insertion is conventionally described in four categories—mucosal, gingival, papillary and papilla-penetrating—following the classification of Placek, Skach and Mrklas [2]. The attachment is not fixed: coronal growth of the alveolar process progressively relocates the insertion in an apical direction, so that more coronal insertions and greater palpable tension are more common in early childhood and become less frequent as the child grows [3]. Gingival-type attachment is the most frequently reported category in pediatric samples, occurring in roughly 45–57% of children across recent cross-sectional series [4,5]. An observed attachment level therefore reflects the developmental stage as much as it reflects individual anatomy, and MLF appearance should be interpreted against the child’s age and dentition rather than classified as an abnormality on the basis of position alone [6].

Any potential anatomical influence of MLF morphology on caries susceptibility would be expected to be most relevant to the adjacent maxillary anterior region, although such an influence should not be interpreted as being restricted exclusively to these teeth [4,7]. A restrictive or prominent attachment could, in theory, limit upper lip elevation and complicate cleaning of the facial–cervical or proximal surfaces of the maxillary incisors, a mechanism first proposed for facial-surface caries in breastfeeding infants [8]. Dental caries, however, is a multifactorial disease in which diet, fluoride exposure, plaque control, brushing frequency, feeding behavior, eruption stage, and socioeconomic status all contribute to risk, and any of these factors could confound an apparent anatomical relationship [9]. Current pediatric guidance does not establish a causal relationship between a hyperplastic maxillary frenulum and caries and does not recommend treatment based on appearance alone [7].

Two recent scoping reviews synthesized MLF classification, diagnosis, and management across broad clinical concerns [10,11]. Neither was designed specifically to map maxillary anterior caries outcomes across pediatric dentitions. A historical clinical report proposed a local substrate-retention mechanism in breastfeeding infants [8], but it did not provide an analyzable comparative patient series. More recent analytical studies have directly evaluated caries outcomes, which supports a focused synthesis of caries in the anatomically adjacent anterior region.

The primary aim of this scoping review was to map studies evaluating MLF morphology or attachment in relation to maxillary anterior dental caries in children and adolescents. Secondary aims were to compare assessment methods, summarize the direction and precision of reported associations where possible, identify methodological and reporting limitations, and define priorities for future research.

## 2. Materials and Methods

### 2.1. Review Design and Protocol

This scoping review followed the updated Joanna Briggs Institute (JBI) methodological guidance [12] and was reported in accordance with the Preferred Reporting Items for Systematic Reviews and Meta-Analyses extension for Scoping Reviews (PRISMA-ScR) [13]. The protocol was registered on the Open Science Framework (OSF; DOI: 10.17605/OSF.IO/ZEBQH). A post-registration amendment, dated 19 August 2026, refined the original early-childhood-caries/primary-dentition scope to maxillary anterior dental caries in pediatric populations. The rationale was to align the outcome with the anatomical region in which an MLF effect could plausibly operate. The amendment broadened eligibility to include primary, mixed, and permanent dentitions while preserving the MLF exposure. All records identified in the initial search conducted on 13 July 2026 were retained and reassessed de novo according to the amended eligibility criteria. Any preliminary screening conducted before the amendment was considered non-final. The amendment was decided and implemented before final study selection and data charting and without using knowledge of the direction, magnitude, or statistical significance of findings from potentially eligible studies to define or apply the amended criteria.

### 2.2. Eligibility Criteria

Eligibility was defined using the Population-Concept-Context framework [12]. The population comprised participants younger than 18 years of age. Primary, mixed, and permanent anterior dentitions were eligible. The concept was MLF morphology, attachment level, attachment type or classification. The context was the maxillary anterior region, and the outcome was dental caries affecting this region, including tooth- or surface-level caries; anterior interproximal caries; maxillary-incisor dmft/dmfs; International Caries Detection and Assessment System (ICDAS)-based anterior lesions [14]; or a clearly reported presence/severity measure for maxillary anterior caries.

Eligible designs were observational analytical studies, including cross-sectional, cohort and case–control studies. Reports were excluded if they did not evaluate a relevant caries outcome, assessed only lingual or mandibular frenula, reported only whole-mouth caries without a separable maxillary anterior outcome, involved adult-only populations, or were reviews, editorials, isolated case reports, or descriptive reports without a systematically defined patient series and analyzable denominator. English- and Turkish-language reports were eligible. No publication-year restriction was applied.

### 2.3. Information Sources and Search Strategy

PubMed/MEDLINE, Scopus and Web of Science Core Collection were initially searched on 13 July 2026 under the original protocol and rerun in full on 19 August 2026 after the protocol amendment. Records identified in the initial search were not evaluated solely according to the original eligibility criteria; all were subsequently reassessed using the amended criteria. The updated strategy combined broad dental-caries and anterior-caries terms with frenum/frenulum terms; the term “early childhood caries” was retained as a sensitivity term because anatomically relevant studies may use that terminology. No age, publication-year, or document-type filters were applied at the search stage. Reference lists of included and potentially relevant reports were also screened.

To improve coverage of the Turkish literature, TR Dizin was searched on 29 August 2026 using Turkish and English combinations of MLF and dental-caries terms. The search yielded eight records: one was the previously identified study by Kınay Taran et al. [4], and seven were new unique records that were excluded at title/abstract screening. The executed TR Dizin query did not return the report by Kılınç et al. [15], which had already been identified through citation searching; this indicates the limited sensitivity of the Turkish-language query itself rather than the report’s absence from Turkish-indexed literature. Complete search strategies are provided in Supplementary Table S1.

The three international databases yielded 97 records (PubMed/MEDLINE 21; Scopus 53; Web of Science Core Collection 23). TR Dizin yielded eight additional records. After the removal of 37 duplicates or known overlaps, 68 unique database records remained for title/abstract screening.

### 2.4. Selection of Sources of Evidence

Following the protocol amendment, two reviewers independently reassessed all 68 unique database records against the amended eligibility criteria, including all records identified in the initial search conducted on 13 July 2026. Final screening decisions were therefore based on the amended criteria rather than the original early-childhood-caries/primary-dentition criteria. Their initial independent decisions were recorded before reconciliation. Agreement was 67/68 (98.5%), with Cohen’s kappa = 0.925. The single disagreement concerned an overview article on oral frenula and was resolved by consensus as Exclude because it did not provide a direct maxillary anterior caries outcome. Sixty-one database records were excluded at the title/abstract level, and seven database reports were sought for retrieval. Citation searching identified three additional reports.

Of the nine unique reports sought for retrieval, the report by Subiksha and Gurunathan [16] could not be retrieved as a full text and was not assigned a full-text eligibility decision. The corresponding author was contacted by email through ResearchGate, but no response was received. The 2015 Kotlow bibliographic record was identified as a duplicate record of the verifiable 2010 Kotlow publication [8] and was therefore not counted as a separate report. Eight retrieved reports underwent independent eligibility assessment, with concordant initial decisions for all eight. The report by Seraj et al. [17] was excluded because children with decay of the maxillary central teeth had been excluded by design, precluding the evaluation of the MLF–anterior caries relationship. Final eligibility assessment thus excluded two retrieved reports and left six analytical studies.

During duplicate data charting, eligibility was rechecked for consistency with the analytical-study criterion. Kotlow’s 2010 report [8], initially retained by both reviewers, did not present a systematically defined analytical patient series, an analyzable denominator by MLF category, or a comparative caries analysis, and was therefore reclassified as background/hypothesis evidence rather than an included source of evidence. Final eligibility assessment thus excluded two retrieved reports and left six analytical studies.

### 2.5. Data Charting

Data charting commenced only after the completion of the amended eligibility assessment and final study selection. A standardized data-charting form captured author and year, country and setting, design, sample size, age, dentition, MLF classification, anterior-caries definition and assessment method, examiner information, statistical approach, effect estimate or precision when available, confounder adjustment, principal findings, and methodological limitations. Both reviewers independently verified charted fields against the primary reports and reconciled discrepancies by discussion. The full text of the article by Kerins et al. [18] was retrieved and reverified against the full text during the final data-charting audit; its four-level Kotlow frenum scale [8], bivariate analyses, and multivariable ordered logistic regression were charted directly from that source.

### 2.6. Synthesis and Numerical Checks

A formal risk-of-bias assessment was not prespecified in the protocol because the objective was evidence mapping rather than the estimation of a pooled causal effect. Instead, methodological features relevant to interpretation were charted study by study, including sampling, exposure and outcome measurement, dentition stage, examiner procedures, confounder handling, analysis unit and statistical reporting. These methodological features were considered explicitly when interpreting the consistency, direction, and potential meaning of the reported associations. In particular, the observational nature of the evidence, the cross-sectional design of all included studies, non-probability or clinic-based sampling, variation in MLF classification systems, differences in caries assessment methods, limited adjustment for potential confounders, and the possibility of measurement and selection bias were taken into account. Accordingly, the findings were interpreted as descriptive and hypothesis-generating rather than as evidence of a causal or independent effect of MLF morphology on maxillary anterior caries. Owing to clinical and methodological heterogeneity, no meta-analysis was planned.

Because formal critical appraisal was not prespecified in the original protocol, an additional item-level appraisal was performed during the revision process in response to reviewer feedback. At the peer-review stage, the six included analytical cross-sectional studies were critically appraised post hoc using the JBI Critical Appraisal Checklist for Analytical Cross-Sectional Studies [19]. Two reviewers (M.A.T. and F.N.) independently completed the appraisal, and any discrepancies were resolved through discussion and consensus. The appraisal was not used to alter the eligibility criteria, study selection, or data charting; rather, it was used to inform the revised interpretation of the evidence. Each study was assessed with respect to participant selection, description of the study subjects and setting, validity and reliability of exposure and outcome measurement, identification and management of confounding factors, and the appropriateness of the statistical analysis. No overall numerical score or predefined cutoff was applied. The item-level appraisal results are presented in Supplementary Table S6.

Where published aggregate 2 × 2 counts permitted, crude odds ratios (ORs) and 95% confidence intervals (CIs) were calculated descriptively using Python 3.11 (Python Software Foundation, Wilmington, DE, USA). For Kınay Taran et al. [4], the primary article reports significance only at a threshold level: *p* < 0.01 in the text and post hoc statements, and 0.01 in the table’s *p*-value column. Recalculation from the published 4 × 2 cell counts yielded Pearson χ^2^ = 26.81, df = 3, *p* < 0.001, consistent with that reported threshold and confirming that the crude ORs were derived from internally coherent cell counts. For Yilgör et al. [20], the reported chi-square result was reproduced, and a fixed-margin exact sensitivity calculation was performed because two expected cell counts were below 5. These checks were descriptive sensitivity analyses of published aggregate data, not adjusted or causal estimates.

### 2.7. AI-Assisted Editorial Support

During manuscript preparation, ChatGPT (GPT-5, OpenAI, San Francisco, CA, USA) was used for language editing, organizational refinement, proofreading, document formatting, and computational cross-checking of descriptive statistics derived from the included reports. ChatGPT was not used to determine study eligibility, conduct record screening, replace independent reviewers, or make final decisions regarding data extraction or interpretation. All screening decisions, eligibility assessments, data-charting decisions, numerical checks, interpretations, and the final manuscript were reviewed and approved by the authors, who take full responsibility for the content of this publication.

## 3. Results

### 3.1. Study Selection

**Figure 1:**
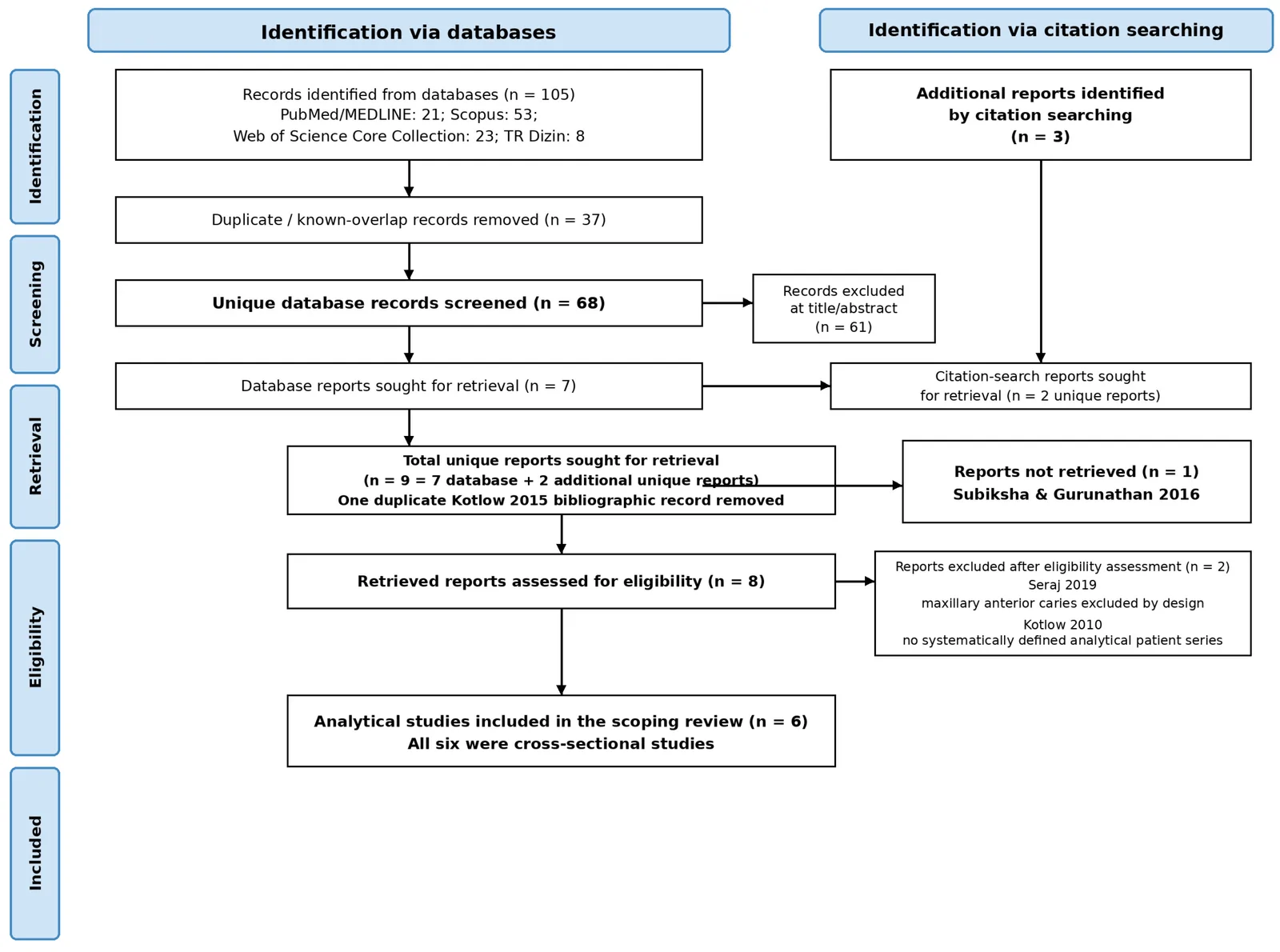
PRISMA-ScR flow diagram of the search and eligibility assessment. International database searches identified 97 records, and the supplementary TR Dizin search identified 8, totaling 105 database records before deduplication. After the removal of 37 duplicate or known-overlap records, 68 unique database records underwent title/abstract screening, and 61 were excluded. Seven database reports and three additional reports identified through citation searching were sought for retrieval. One duplicate bibliographic record of the Kotlow 2010 publication was removed before retrieval assessment [8]. One report was not retrieved [16], eight retrieved reports underwent eligibility assessment, two were excluded after final eligibility review [8,17], and six analytical cross-sectional studies were included in the final scoping review.

The final database set comprised 105 records. After the removal of 37 duplicates or known overlaps, 68 unique database records were screened. Initial title/abstract agreement was 67/68 (98.5%; Cohen’s kappa = 0.925); 61 records were excluded, and seven database reports were sought. Citation searching added three further reports. During verification, the 2015 Kotlow record was identified as a duplicate of the verifiable 2010 Kotlow publication [8] and was not counted as a separate report. Thus, nine unique reports were sought for retrieval. One report, Subiksha and Gurunathan [16], could not be retrieved; eight retrieved reports underwent eligibility assessment, and two were excluded after final eligibility review: Seraj et al. [17], because relevant anterior caries had been excluded by design, and Kotlow 2010 [8], because it did not meet the analytical patient-series criterion. Six studies were included in the final evidence set, as shown in Figure 1.

### 3.2. Characteristics of Included Studies

The final evidence set comprised six analytical cross-sectional studies published between 2014 and 2026 from Türkiye, India and the United States [4,5,15,18,20,21]. Sample sizes ranged from 92 to 500 participants. Ages ranged from 6 months to 16 years, spanning primary, mixed and permanent anterior dentitions. Most samples were clinic- or practice-based rather than representative of the general population. Study characteristics are summarized in Table 1.

### 3.3. Variation in Frenulum and Caries Assessment

All six included studies operationalized maxillary frenum insertion using four positional categories corresponding to mucosal or most superior, gingival, papillary, and papilla-penetrating or most inferior attachment. Five studies used the Placek framework directly or cited the same four-category system under the bibliographic label ‘Mirko’ [4,5,15,20,21]. Kerins et al. [18] used the Kotlow four-point scale [8]; in the full text, Classes I–IV were explicitly defined as mucosal/most superior, gingival, papillary and papilla-penetrating/most inferior, respectively. Kılınç et al. [15] additionally assessed frenulum morphology using a modified Sewerin system. The exposure definition was therefore largely shared across the evidence base, so the inconsistent caries findings are unlikely to be explained by fundamentally different insertion-position categories.

Caries ascertainment, by contrast, varied substantially: anterior approximal caries [15], maxillary-incisor dmft categories [4], binary anterior caries [5,20], ICDAS-based lesion severity [21] and graded anterior caries [18]. These outcomes represent different lesion stages, surfaces and developmental contexts and were not suitable for quantitative pooling.

### 3.4. Findings on MLF Type and Maxillary Anterior Caries

Unless otherwise indicated, the ORs presented in this section are crude ORs recalculated by the review authors from published aggregate counts. Adjusted estimates are identified explicitly as originating from the primary study.

Kılınç et al. found no statistically significant relationship between MLF type and anterior approximal caries (*p* = 0.521) [15]. Kınay Taran et al. reported lower maxillary-primary-incisor dmft in the mucosal group and higher caries experience in the papillary group, with the overall comparison reported as *p* < 0.01 [4]. From the published cell counts, the review authors recalculated crude ORs for dmft ≥ 1 relative to mucosal attachment as 2.26 (95% CI 1.05–4.88) for gingival, 15.42 (95% CI 4.60–51.74) for papillary and 4.81 (95% CI 1.58–14.64) for papilla-penetrating attachment. Recalculation of the published 4 × 2 table yielded *p* < 0.001, consistent with the primary report’s threshold-level significance. The outcome definitions, reported results, review-author calculations, and methodological constraints are presented in Table 2.

Yilgör et al. found that attachment distribution differed between children with and without maxillary anterior caries (χ^2^ = 6.982; *p* = 0.03) [20]. Using gingival attachment as the reference, the review authors recalculated crude ORs of 3.33 (95% CI 1.14–9.72) for mucosal attachment and 4.00 (95% CI 0.75–21.38) for the combined papillary/papilla-penetrating category. Because two expected cell counts were below 5, an exact fixed-margin sensitivity calculation was performed and yielded *p* = 0.030, supporting the reported overall association. The direction was nevertheless non-monotonic: the mucosal type was also more frequent among caries cases, arguing against a simple gradient in which a more coronal attachment implies greater caries risk.

The crude ORs derived for Kınay Taran et al. [4] and Yilgör et al. [20] were calculated from unadjusted aggregate data and should not be interpreted as independent effects of MLF attachment. Their magnitude may have been influenced by age, dentition stage, feeding behavior, oral-hygiene practices, fluoride exposure, socioeconomic factors, or other unmeasured determinants of childhood caries. Accordingly, the larger crude estimates were not given disproportionate interpretive weight in the overall synthesis.

Kalaivanan et al. reported more affected anterior teeth in the papillary group (*p* = 0.01) and more non-cavitated lesions in the gingival group (*p* = 0.04), while cavitated lesion counts did not differ significantly (*p* = 0.182) [21]. The study enrolled children with anterior early childhood caries and therefore primarily addressed disease severity rather than disease occurrence. The report stated a total sample size of *n* = 131, whereas the tabulated sex and MLF-category counts each summed to 130. This discrepancy may affect the reported denominators, group proportions, and the precision of the statistical comparisons; however, its quantitative impact could not be determined from the published information. The discrepancy was therefore treated as a reporting limitation, and no participant-level OR was derived from this study.

Tokgöz Kaplan and Gökcek Taraç, in the largest included sample (*n* = 500) spanning primary (*n* = 159), mixed (*n* = 282) and permanent (*n* = 59) dentitions, reported no statistically significant association between anterior caries and MLF type in the full-text Results (*p* = 0.058) [5], despite abstract wording implying a significant relationship; the full-text result was used in this review. No odds ratio or prevalence ratio was reported, and the published data did not permit an independent recalculation. The near-null finding in the largest and developmentally most diverse study is consistent with the heterogeneous findings across the evidence base but does not by itself resolve the observed inconsistency.

Kerins et al. found no bivariate association between frenum classification and ICDAS caries severity (Kruskal–Wallis *p* = 0.32) [18]. In the primary study’s multivariable ordered logistic regression controlling for age and payer source, none of the MLF categories differed significantly from Class I: Class II OR 1.37 (95% CI 0.72–2.61; *p* = 0.33), Class III OR 0.92 (95% CI 0.50–1.70; *p* = 0.81), and Class IV OR 1.85 (95% CI 0.98–3.49; *p* = 0.06). Although the point estimate for Class IV was numerically higher, the association was not statistically significant, and the confidence interval was wide. The finding should therefore be interpreted as imprecise and inconclusive. Age remained associated with greater caries severity (OR 1.06 per month, 95% CI 1.04–1.09; *p* < 0.001).

Taken together, the included studies did not demonstrate a consistent or reproducible anatomical risk gradient; apparent differences in direction should be interpreted in light of the heterogeneity described in Section 3.3.

### 3.5. Methodological Limitations and Evidence Gaps

All six included studies were cross-sectional, so the temporality between MLF anatomy, functional restriction, plaque persistence, and incident caries could not be established. Clinic- or practice-based sampling was used throughout, with no population-representative sample. Although all six studies used four positional insertion categories with closely corresponding anatomical meanings, age and dentition stage varied markedly, and caries outcomes ranged from approximal lesions and dmft categories to ICDAS-based severity. Only Kerins et al. [18] reported a multivariable MLF model, controlling for age and payer source; diet, oral-hygiene practices, and fluoride exposure were not measured, participating dentists were trained but not calibrated, and the pilot sample yielded imprecise category-specific estimates. The remaining studies generally lacked adjusted MLF effect estimates. The principal evidence gaps and priorities for future research are summarized in Table 3.

The JBI critical appraisal (Supplementary Table S6) was consistent with these observations: only Kerins et al. reported strategies to address confounding (item 6), confounding factors were not clearly identified in three studies (item 5), and the validity and reliability of outcome measurement were unclear in four studies (item 7).

## 4. Discussion

### 4.1. Principal Findings

Six analytical cross-sectional studies evaluated MLF morphology or attachment in relation to maxillary anterior caries in pediatric populations [4,5,15,18,20,21]. The evidence was heterogeneous, and the reported directions of association were not consistent across studies. This apparent directional inconsistency should be interpreted in the context of substantial heterogeneity in caries definitions, age groups, dentition stages, participant selection, and study populations. Selected bivariate differences were reported by Kınay Taran et al., Yilgör et al. and Kalaivanan et al. [4,20,21], but these did not form a consistent anatomical gradient and depended on the outcome definition. Kılınç et al., Tokgöz Kaplan and Gökcek Taraç, and Kerins et al. reported null or near-null overall associations [5,15,18], although Kerins et al.’s adjusted analysis yielded an imprecise estimate for the most coronal attachment class, with no MLF category differing significantly from the reference class. No study established an adjusted prospective effect of MLF anatomy on incident anterior caries.

The broader MLF scoping reviews published in 2024 and 2025 focused on classification, diagnosis, and treatment across multiple clinical indications [10,11]. The present review differs by restricting the outcome to anatomically relevant maxillary anterior caries and by incorporating newer caries-focused studies, including those by Kalaivanan et al., Tokgöz Kaplan and Gökcek Taraç, and Kerins et al. [5,18,21]. This focus distinguishes local anterior evidence from broad whole-mouth early-childhood-caries terminology.

### 4.2. Sources of Inconsistency

As shown in Section 3.3, the MLF insertion-position classification was highly comparable across the evidence base, so the inconsistent caries findings are more plausibly related to other design features than to fundamentally different exposure categories. Studies measured different caries constructs, including approximal lesions, maxillary-incisor dmft categories, binary anterior caries, ICDAS lesion counts and graded severity, across different ages and dentitions. A relationship observed for non-cavitated lesions in the primary dentition need not persist for cavitated disease or in the mixed and permanent dentitions.

Confounding is a second major limitation. Nocturnal feeding, frequency of fermentable-carbohydrate exposure, brushing assistance, fluoride availability, visible plaque, eruption timing, age, parental education and household resources can all influence anterior caries. Kerins et al. [18] adjusted for age and payer source and still found no significant association between MLF class and caries severity, but diet, oral-hygiene practices and fluoride exposure were not collected, and the category-specific estimates were imprecise. The remaining studies generally did not report adjusted MLF effect estimates. Observed bivariate differences may therefore reflect age structure, clinic selection, behavior or residual confounding rather than an independent anatomical effect. In particular, the relatively large crude ORs calculated for some attachment categories should be interpreted cautiously and should not be considered stronger evidence than the null or near-null findings from other studies. Because these estimates were unadjusted, their magnitude may reflect residual or uncontrolled confounding rather than a specific independent effect of MLF morphology.

A third consideration is statistical power. The evidence base contains only one adjusted analysis, drawn from a pilot study, and its category-specific estimates were imprecise, with wide confidence intervals. A null result in a categorical subgroup analysis of this size should not be equated with demonstrated absence of an effect; it is equally compatible with a modest effect that the study was not designed to detect with adequate precision.

Finally, an insertion-site category is not equivalent to functional restriction. Children with the same papillary or gingival attachment can differ in frenulum thickness, elasticity, tension, lip elevation, vestibular depth and cleaning difficulty. Future exposure definitions should therefore combine the common anatomical classification with reproducible functional measures.

### 4.3. Biological Plausibility and the Historical Hypothesis

A biologically plausible pathway remains that a genuinely restrictive and tense MLF could limit upper lip elevation and make cleaning of facial–cervical or proximal maxillary anterior surfaces more difficult. Kotlow’s historical report proposed such a mechanism in breastfeeding infants [8], but it was retained as background rather than as included analytical evidence because it did not provide a systematically defined comparative patient series. Biological plausibility thus remains a hypothesis, not a finding, and testing it will require measuring functional restriction, plaque accumulation, and incident caries within the same longitudinal study.

### 4.4. Clinical Implications

MLF inspection may form part of a comprehensive pediatric oral examination, particularly when caregivers report difficulty elevating the upper lip or cleaning the maxillary anterior cervical region. As part of such a clinical assessment, clinicians may consider lip mobility, frenulum tension, blanching, vestibular depth, visible plaque, brushing technique, dietary exposures, fluoride exposure, and developmental stage alongside the child’s overall caries-risk profile. This should be understood as a suggested comprehensive assessment framework rather than as an evidence-based clinical protocol, because these factors were not evaluated together or tested as a combined predictive model in the included studies. MLF attachment should not be used as a stand-alone caries risk marker.

No included study tested caries prevention after frenectomy. Therefore, current evidence does not support recommending frenectomy solely for the purpose of preventing maxillary anterior caries; this reflects insufficient evidence of benefit rather than evidence that frenectomy is ineffective for caries prevention. This conclusion does not address other legitimate functional, feeding, periodontal/mucogingival, orthodontic or selected speech-related indications. Surgical decisions should remain based on documented functional indications and age-appropriate multidisciplinary assessment rather than on caries prevention alone [7].

### 4.5. Strengths and Limitations of the Review

Strengths include a transparent post-registration protocol amendment, the rerunning of the international database searches, a supplementary TR Dizin search, auditable duplicate screening with the preservation of initial decisions, duplicate data charting and explicit numerical checks of review-author-derived statistics. TR Dizin rediscovered one included study but did not return the report by Kılınç et al. under the executed query [15]; this platform-specific retrieval limitation is reported rather than presented as comprehensive coverage, and it indicates that citation searching remained necessary for the complete identification of the Turkish-language evidence.

The JBI appraisal indicated that the main limitations across the included studies concerned the cross-sectional design, clinic- or practice-based sampling, incomplete control of important confounding factors, and variation in the measurement of MLF morphology and caries outcomes. These limitations reduce confidence in interpreting the reported associations as independent effects of MLF morphology.

Limitations include one report that could not be retrieved as full texts [16] and the restriction to English- and Turkish-language reports. The non-retrieval of this report may have introduced retrieval bias, and language bias cannot be excluded; the direction and magnitude of either bias could not be assessed because the potentially relevant reports were not available for full evaluation. In addition, critical appraisal was not prespecified in the protocol. The item-level JBI appraisal was added post hoc and did not yield an overall risk-of-bias score or judgment; study-level internal validity was therefore characterized descriptively rather than graded, and the strength of the mapped associations should be interpreted cautiously (Supplementary Table S6). All included studies were cross-sectional, and the evidence base showed variation in participant selection, dentition stage, MLF classification, caries assessment, examiner procedures, and adjustment for potential confounding factors. These differences limit direct comparison across studies and make it difficult to determine whether observed associations reflect a specific effect of MLF morphology or the influence of coexisting behavioral, dietary, oral-hygiene, socioeconomic, and developmental factors. Even the most extensively adjusted included study controlled only for age and payer source, did not measure diet, oral-hygiene practices or fluoride exposure, and produced imprecise category-specific estimates [18]. Reporting discrepancies in some primary studies further limited causal and quantitative interpretation. The available studies generally do not establish temporality or causality. Therefore, the findings should be regarded as descriptive and hypothesis-generating, and MLF morphology should not be considered an established independent risk factor for maxillary anterior caries. Funding sources of the primary studies were not systematically charted and are not reported here. The conclusions of this review should therefore be read as a demonstration that the current evidence base is inadequate to support a clinical inference, rather than as a demonstration that no association exists.

## 5. Conclusions

Evidence linking MLF morphology or attachment to maxillary anterior dental caries in children is limited and heterogeneous, with no consistent association demonstrated across studies. Apparent differences in the direction of association should be interpreted in light of the substantial variation in caries definitions, age groups, dentition stages, participant selection, and study populations. Across six analytical cross-sectional studies, selected bivariate differences were counterbalanced by null or near-null overall findings. Only one study reported a multivariable ordered logistic regression; after controlling for age and payer source, no MLF class differed significantly from the reference class, although the category-specific estimates were imprecise. No longitudinal study established temporality. Current evidence therefore does not support using MLF attachment type as a stand-alone caries risk marker or recommending frenectomy solely for the purpose of preventing maxillary anterior caries. For frenectomy, this reflects insufficient evidence of benefit rather than evidence that it is ineffective for caries prevention. Adequately powered prospective studies that combine the shared anatomical classification with reproducible functional measures and prespecified confounder control are required before any clinical inference can be drawn.

## Figures and Tables

**Table 1:** Characteristics and principal findings of included analytical studies.

Study	Setting/Design	Sample and Age	MLF Assessment	Anterior-Caries Assessment	Main Finding
Kılınç et al. 2014 [15]	Türkiye (İzmir); 3 pediatric dental clinics; cross-sectional	*n* = 403; 3–16 y	Placek four-category attachment system (reported as Mirko) + modified Sewerin morphology	Approximal caries of maxillary anterior teeth	No MLF–caries relationship (*p* = 0.521).
Kınay Taran et al. 2023 [4]	Türkiye (İstanbul); Bezmialem Vakıf University pediatric dentistry clinic; cross-sectional	*n* = 214; 3–6 y	Placek four-category attachment system	WHO criteria; maxillary-primary-incisor dmft < 1 vs. ≥1	Papillary attachment showed the strongest crude association with dmft ≥ 1; overall difference reported as *p* < 0.01.
Yilgör et al. 2025 [20]	Türkiye (Erzurum); Atatürk University pediatric dentistry clinic; cross-sectional	*n* = 92; 24–71 mo	Placek four-category attachment system	WHO visual-tactile examination; maxillary anterior caries presence	Attachment distribution differed by caries status (χ^2^ = 6.982; *p* = 0.03), with a non-monotonic direction.
Kalaivanan et al. 2025 [21]	India; pediatric dentistry department and private practice; exploratory cross-sectional	reported *n* = 131; tabulated counts = 130; <6 y	Placek four-category attachment system (cited by the primary report as Mirko et al.)	ICDAS II; affected teeth; non-cavitated/cavitated lesions	Papillary type had more affected anterior teeth; gingival type had more non-cavitated lesions; cavitated lesions did not differ significantly.
Tokgöz Kaplan and Gökcek Taraç 2025 [5]	Türkiye (Karabük); Karabük University pediatric dentistry clinic; cross-sectional	*n* = 500; 3–14 y	Placek four-category attachment system (reported as Mirko)	Anterior-region caries present/absent	Full-text Results: no statistically significant association (*p* = 0.058).
Kerins et al. 2026 [18]	USA (South Texas); practice-based network; pilot cross-sectional	*n* = 319; 6–36 mo	Kotlow four-level scale [8]: I mucosal/most superior; II gingival; III papillary; IV papilla-penetrating/most inferior	ICDAS score on facial surfaces of primary maxillary anterior teeth; highest score recorded	No bivariate association (*p* = 0.32); no MLF class differed significantly from Class I after adjustment for age and payer source, although the Class IV estimate was imprecise and not statistically significant.

ICDAS: International Caries Detection and Assessment System; MLF: maxillary labial frenulum; WHO: World Health Organization.

**Table 2:** Outcome definitions and quantitative findings of the included studies.

Study	Outcome/Comparison	Primary Result	Review-Author Estimate/Check	Adjustment	Interpretive Constraint
Kılınç 2014 [15]	Anterior approximal caries vs. attachment type	*p* = 0.521	No OR/PR reported	No adjusted MLF effect reported	Broad age/dentition range; null bivariate result.
Kınay Taran 2023 [4]	Maxillary-incisor dmft < 1 vs. ≥1	Reported *p* < 0.01	Crude OR vs. mucosal: gingival 2.26 (1.05–4.88); papillary 15.42 (4.60–51.74); papilla-penetrating 4.81 (1.58–14.64). Recalculated Pearson *p* < 0.001, consistent with the reported threshold.	No adjusted MLF effect reported	Cross-sectional clinic sample; residual confounding.
Yilgör 2025 [20]	Anterior caries presence vs. attachment distribution	χ^2^ = 6.982; *p* = 0.03	OR vs. gingival: mucosal 3.33 (1.14–9.72); papillary/papilla-penetrating 4.00 (0.75–21.38); exact sensitivity *p* = 0.030.	No adjusted MLF effect reported	Sparse cells; non-monotonic direction.
Kalaivanan 2025 [21]	Affected teeth; non-cavitated/cavitated lesions	*p* = 0.01; *p* = 0.04; *p* = 0.182	No participant-level OR derived	No comprehensive adjustment reported	Severity-only sample; stated n = 131 but table totals = 130.
Kaplan and Taraç 2025 [5]	Anterior caries present/absent	Full-text *p* = 0.058	No OR/PR reported	No adjusted MLF effect reported	Abstract/full-text discrepancy.
Kerins 2026 [18]	ICDAS anterior caries severity across Kotlow Classes I–IV	Bivariate MLF *p* = 0.32; age *p* < 0.001; payer source *p* = 0.31	Adjusted ordered logistic regression vs. Class I: II OR 1.37 (0.72–2.61), *p* = 0.33; III OR 0.92 (0.50–1.70), *p* = 0.81; IV OR 1.85 (0.98–3.49), *p* = 0.06. Age OR 1.06 per month (1.04–1.09), *p* < 0.001.	Adjusted for age and payer source	Pilot practice-based sample; dentists trained but not calibrated; diet, oral hygiene and fluoride exposure not measured; Class IV estimate was imprecise, with a wide confidence interval, and was not statistically significant.

ICDAS: International Caries Detection and Assessment System; MLF: maxillary labial frenulum; OR: odds ratio; PR: prevalence ratio. For Kınay Taran and Yilgör, ORs are crude review-author calculations from published aggregate counts; for Kerins, adjusted ORs are reported directly from the primary multivariable ordered logistic regression.

**Table 3:** Evidence gaps and priorities for future research.

Domain	Current Evidence	Implication
Temporality	All six included studies were cross-sectional.	Prospective cohorts should measure incident anterior lesions.
MLF measurement	All six studies used four positional insertion categories with closely corresponding anatomy; five used Placek or its ‘Mirko’ bibliographic label [2] and Kerins used the Kotlow I–IV scale [8].	Retain common anatomical categories but add functional measures: lip elevation, tension, blanching, vestibular depth and cleanability.
Caries outcome	Approximal caries, dmft categories, binary caries, ICDAS lesions and severity grades varied.	Use standardized tooth- and surface-level anterior outcomes.
Development	Participants ranged from infancy to adolescence across dentitions.	Stratify by age, eruption status and dentition.
Confounding	Only one study reported an adjusted MLF model; behavioral and socioeconomic determinants were otherwise incompletely controlled.	Use prespecified multivariable models and adjusted estimates with 95% CIs.
Sampling	Predominantly clinic/practice-based samples from three countries.	Use multicenter/community-based cohorts.
Statistical power	The only adjusted analysis produced imprecise category-specific estimates with wide confidence intervals.	Power studies for a modest anatomical effect rather than reporting only null significance tests.

CI: confidence interval; MLF: maxillary labial frenulum.

## Data Availability

The protocol and dated post-registration amendment are available through the associated Open Science Framework project (https://doi.org/10.17605/OSF.IO/ZEBQH). Search exports, deduplication records, independent screening files, reconciliation records, duplicate data-charting records and numerical audit files are retained with the review records. No participant-level dataset was generated.

## Supplementary Materials

The following supporting information can be downloaded at: https://www.mdpi.com/article/10.3390/children13101340/s1, Supplementary Table S1 presents the complete search strategies and supplementary platform searches; Supplementary Table S2 presents full-text retrieval and final eligibility dispositions; Supplementary Table S3 presents the extended study-level data chart; Supplementary Table S4 presents numerical and reporting consistency checks; Supplementary Table S5 provides the PRISMA-ScR reporting-location map; and Supplementary Table S6 presents the item-level JBI critical appraisal of the six included analytical cross-sectional studies. A completed PRISMA-ScR checklist is provided as a separate submission file.
