# Supplementary material for: Maxillary Labial Frenulum Morphology and Maxillary Anterior Dental Caries in Children: A Scoping Review
Source: Children (Basel). 2026 Oct 2;13(10):1340. doi: 10.3390/children13101340 (PMC13703020; doi:10.3390/children13101340)
Supplement: Supplementary file 1 [file children-13-01340-s001.zip › Children_PRISMA_ScR_Checklist_updated.pdf]

# PRISMA-ScR Checklist

## Maxillary Labial Frenulum Morphology and Maxillary Anterior Dental Caries in Children: A Scoping Review

Completed PRISMA-ScR reporting checklist. Item descriptions are condensed from the PRISMA-ScR checklist for submission use. Locations are given by manuscript section rather than page number because pagination will be assigned during production.

Checklist source: Tricco, A.C.; Lillie, E.; Zarin, W.; O’Brien, K.K.; Colquhoun, H.; Levac, D.; et al. PRISMA extension for scoping reviews (PRISMA-ScR): Checklist and explanation. Ann. Intern. Med. 2018, 169, 467–473.

| Section | Topic | Item | Reporting requirement | Reported in |
|---|---|---|---|---|
| TITLE | Title | 1 | Identify the report as a scoping review. | Title page; title includes “a scoping review”. |
| ABSTRACT | Structured summary | 2 | Summarize background/objectives, eligibility, information sources, charting methods, main results, and conclusions. | Structured Abstract with Background/Objectives, Methods, Results, and Conclusions. |
| INTRODUCTION | Rationale | 3 | Explain the rationale and why a scoping review approach was appropriate. | Section 1, Introduction. |
| INTRODUCTION | Objectives | 4 | State the review objectives and key conceptual elements. | Section 1, final paragraph. |
| METHODS | Protocol and registration | 5 | State whether a protocol exists, where it can be accessed, and registration information. | Section 2.1; OSF registration and dated post-registration amendment. |
| METHODS | Eligibility criteria | 6 | Specify inclusion/exclusion criteria and important restrictions. | Section 2.2. |
| METHODS | Information sources | 7 | Describe all information sources and the most recent search dates. | Section 2.3; Supplementary Table S1. |
| METHODS | Search | 8 | Present reproducible electronic search strategies and limits. | Section 2.3; Supplementary Table S1. |
| METHODS | Selection of sources | 9 | Describe how sources were screened and selected. | Section 2.4. |
| METHODS | Data charting process | 10 | Describe the charting process, independent verification, and discrepancy resolution. | Section 2.5. |
| METHODS | Data items | 11 | List and define variables and information sought from each source. | Section 2.5; Supplementary Table S3. |
| METHODS | Critical appraisal | 12 | If performed, describe appraisal methods; otherwise explain why it was not undertaken. | Section 2.6; item-level JBI appraisal of six included studies was conducted by two independent reviewers during revision; no overall score or cutoff was applied. |
| METHODS | Synthesis of results | 13 | Describe how charted data were handled and synthesized. | Section 2.6. |
| RESULTS | Selection of sources | 14 | Report numbers screened, assessed, and included, with reasons for exclusions and a flow diagram. | Section 3.1; Figure 1; Supplementary Table S2. |
| RESULTS | Characteristics of sources | 15 | Present key characteristics of each included source and cite the sources. | Section 3.2; Table 1; Supplementary Table S3. |
| RESULTS | Critical appraisal within sources | 16 | If formal appraisal was performed, present the results. | Item-level JBI appraisal results are presented in Supplementary Table S6 and summarized in Discussion, Section 4.5. The appraisal informed interpretation and did not affect eligibility. |
| RESULTS | Results of individual sources | 17 | Present relevant charted findings for each included source. | Section 3.4; Table 2; Supplementary Table S3. |
| RESULTS | Synthesis of results | 18 | Summarize and display the charted evidence in relation to the objectives. | Sections 3.3–3.5; Tables 2–3; Supplementary Table S4. |
| DISCUSSION | Summary of evidence | 19 | Summarize main results, relevance, and implications. | Sections 4.1–4.4. |

| Section | Topic | Item | Reporting requirement | Reported in |
|---|---|---|---|---|
| DISCUSSION | Limitations | 20 | Discuss limitations of the review process and available evidence. | Section 4.5; unretrieved reports, language restriction, cross-sectional designs, post hoc JBI appraisal, and primary-study reporting inconsistencies. |
| CONCLUSIONS | Conclusions | 21 | Provide an interpretation consistent with objectives and identify implications. | Section 5. |
| FUNDING | Funding sources | 22 | Describe funding of the scoping review and, if relevant, funding of included sources. | Funding statement; primary-study funding was not systematically charted. |
