# Supplementary material for: Maxillary Labial Frenulum Morphology and Maxillary Anterior Dental Caries in Children: A Scoping Review
Source: Children (Basel). 2026 Oct 2;13(10):1340. doi: 10.3390/children13101340 (PMC13703020; doi:10.3390/children13101340)
Supplement: Supplementary file 1 [file children-13-01340-s001.zip › Supplementary Tables S1-S6.pdf]

Supplementary Materials

Maxillary Labial Frenulum Morphology and Maxillary Anterior Dental Caries in Children: A Scoping Review

Mehmet Ali Tuna, Faezeh Naderloo, Ufuk Kesmez, Ayşegül Aydın Tanas, Sera Simsek Derelioğlu

Supplementary Table S1. Complete search strategies

Source	Search date	Search strategy / implementation	Records
PubMed/MEDLINE	19 August 2026	(("Dental Caries"[Mesh] OR "early childhood caries"[Title/Abstract] OR "dental caries"[Title/Abstract] OR caries[Title/Abstract] OR carious[Title/Abstract] OR "tooth decay"[Title/Abstract] OR "anterior caries"[Title/Abstract] OR "anterior dental caries"[Title/Abstract] OR "anterior tooth caries"[Title/Abstract]) AND ("Frenum"[Mesh] OR frenum[Title/Abstract] OR frenulum[Title/Abstract] OR frenal[Title/Abstract] OR "labial frenum"[Title/Abstract] OR "labial frenulum"[Title/Abstract] OR "maxillary frenum"[Title/Abstract] OR "maxillary frenulum"[Title/Abstract] OR "maxillary labial frenum"[Title/Abstract] OR "maxillary labial frenulum"[Title/Abstract]))	21
Scopus	19 August 2026	TITLE-ABS-KEY("early childhood caries" OR "dental caries" OR caries OR carious OR "tooth decay" OR "anterior caries" OR "anterior dental caries" OR "anterior tooth caries") AND TITLE-ABS-KEY(frenum OR frenulum OR frenal OR "labial frenum" OR "labial frenulum" OR "maxillary frenum" OR "maxillary frenulum" OR "maxillary labial frenum" OR "maxillary labial frenulum")	53
Web of Science Core Collection	19 August 2026	TS=("early childhood caries" OR "dental caries" OR caries OR carious OR "tooth decay" OR "anterior caries" OR "anterior dental caries" OR "anterior tooth caries") AND TS=(frenum OR frenulum OR frenal OR "labial frenum" OR "labial frenulum" OR "maxillary frenum" OR "maxillary frenulum" OR "maxillary labial frenum" OR "maxillary labial frenulum")	23
TR Dizin	29 August 2026	Advanced search using Turkish and English combinations of MLF/frenulum terms and dental-caries terms. No year or document-type restriction was applied. The complete Turkish and English term blocks are reported in the manuscript Methods section and were executed as two combined concept blocks.	8
Citation searching	After database screening	Reference lists of included and potentially relevant reports were screened for additional potentially eligible reports. Three bibliographic records were identified; one duplicate Kotlow record was removed during verification, leaving three citation-search reports in the final retrieval set.	3

The final database and citation-search sets were assessed together during full-text retrieval and final eligibility assessment.

Supplementary Table S2. Full-text retrieval and final eligibility dispositions

Report	Route	Retrieval	Initial dual decision	Final disposition	Primary reason
Kerins et al. 2026	Database	Retrieved	Include / Include	Include	Met the amended criteria; direct maxillary anterior caries outcome.
Yilgör et al. 2025	Database	Retrieved	Include / Include	Include	Direct maxillary anterior caries outcome related to MLF attachment.
Kalaivanan et al. 2025	Database	Retrieved	Include / Include	Include	Anterior caries severity evaluated by MLF type.
Kınay Taran et al. 2023	Database	Retrieved	Include / Include	Include	Maxillary-primary-incisor caries evaluated by MLF type.
Seraj et al. 2019	Database	Retrieved	Exclude / Exclude	Exclude	Children with maxillary central-tooth decay were excluded by design.
Kotlow 2010	Database	Retrieved	Include / Include	Exclude after audit	No analytical patient series or analyzable MLF-category denominator; retained as background.
Subiksha and Gurunathan 2016	Citation searching	Not retrieved	Exclude / Exclude	Not retrieved	Potential relevance could not be confirmed from inaccessible full text.
Kılınç et al. 2014	Citation searching	Retrieved	Include / Include	Include	Direct anterior approximal caries outcome.
Tokgöz Kaplan and Gökcek Taraç 2025	Citation searching	Retrieved	Include / Include	Include	Direct anterior caries outcome; full-text $p = 0.058$ used.

The final evidence set comprised six analytical cross-sectional studies. One report could not be retrieved, and two retrieved reports were excluded after final eligibility ass essment. The 2015 Kotlow bibliographic record was removed as a duplicate of the verifiable 2010 report.

Supplementary Table S3. Extended study-level data chart

Part A. Study design, population, MLF assessment, and caries outcome

Study	Design / sample	Age / dentition	MLF assessment	Caries outcome
Kılınç et al. 2014	Cross-sectional; n = 403; three pediatric dental clinics in İzmir	3–16 years; primary, mixed, permanent	Mirko classification and modified Sewerin morphology	Anterior approximal caries
Kınay Taran et al. 2023	Cross-sectional; n = 214; university clinic	3–6 years; primary	Placek four-category	Maxillary-primary-incisor dmft <1 vs. ≥1
Yilgör et al. 2025	Cross-sectional; n = 92	24–71 months; primary	Placek; standardized photographs	Presence of maxillary anterior caries
Kalaivanan et al. 2025	Exploratory cross-sectional; reported n = 131	<6 years; primary	Mirko four-category	ICDAS II severity; affected teeth; cavitation
Tokgöz Kaplan and Gökcek Taraç 2025	Cross-sectional; n = 500; university-based	3–14 years; primary, mixed, permanent	Mirko classification	Anterior caries present/absent
Kerins et al. 2026	Pilot cross-sectional; n = 319; practice-based network	6–36 months; primary	Four attachment classes	ICDAS-based anterior caries grade

Part B. Study-level statistics and interpretive limitations

Study	Statistics	Interpretive limitations
Kılınç et al. 2014	p = 0.521	Broad age/dentition range; no adjusted effect.
Kınay Taran et al. 2023	Reported p = 0.01 or p < 0.01; review χ^2 = 26.81, p < 0.001; crude ORs	Reported p-value inconsistent with table counts; no adjusted effect.
Yilgör et al. 2025	χ^2 = 6.982, p = 0.030; exact p = 0.030; crude ORs	Sparse cells; non-monotonic direction; no adjusted effect.
Kalaivanan et al. 2025	Affected teeth p = 0.01; non-cavitated p = 0.04; cavitated p = 0.182	Reported n = 131; sex and MLF tables sum to 130.
Tokgöz Kaplan and Gökcek Taraç 2025	Full-text p = 0.058	Abstract/full-text discordance; no adjusted effect.
Kerins et al. 2026	Attachment p = 0.32; age p < 0.001; insurance p = 0.31; adjusted ordered-logistic model	Pilot/practice-based; limited behavioral adjustment; imprecise estimates.

Abbreviations: ICDAS, International Caries Detection and Assessment System; MLF, maxillary labial frenulum; OR, odds ratio; dmft, decayed, missing, and filled primary teeth. Table S3 is intentionally divided into Parts A and B to preserve complete text and prevent column clipping.

Supplementary Table S4. Numerical and reporting consistency checks

Study or report	Issue	Review-author check	Handling in review
Kınay Taran 2023	Primary report gives $p = 0.01/p < 0.01$ for published 4 × 2 table.	Pearson $\chi^2 = 26.81$, df = 3, $p = 0.00000645$ (<0.001).	Both values disclosed; crude ORs based on published cell counts.
Yilgör 2025	Two expected cells below 5 in 3 × 2 table.	Pearson $\chi^2 = 6.982$, $p = 0.0305$; exact $p = 0.02984$.	Exact sensitivity calculation supports $p \approx 0.03$; sparse-cell uncertainty noted.
Kalaivanan 2025	Reported n = 131; sex and MLF tables sum to 130.	No participant-level OR calculated from inconsistent denominators.	n = 131 retained; discrepancy explicitly reported.
Tokgöz Kaplan 2025	Abstract implies significance; full text reports $p = 0.058$.	Full-text result used as controlling result.	Review reports $p = 0.058$ and notes discrepancy.
Kotlow 2010	No systematically defined analytical series or analyzable denominator.	Eligibility rechecked during duplicate charting.	Excluded from analytical evidence; retained as background.

Supplementary Table S5. PRISMA-ScR reporting-location map

PRISMA-ScR area	Location in manuscript / supplement
Title / identification as scoping review	Title page
Structured summary	Abstract: Background/Objectives, Methods, Results, Conclusions
Rationale and objectives	Introduction
Protocol and registration	Methods 2.1; OSF registration and dated amendment
Eligibility criteria	Methods 2.2
Information sources and search	Methods 2.3; Supplementary Table S1
Selection process	Methods 2.4
Data charting	Methods 2.5
Critical appraisal / methodological handling	Methods 2.6; Results 3.5; Supplementary Tables S3–S4, S6
Synthesis	Methods 2.6; Results 3.3–3.5
Selection results	Results 3.1; Figure 1; Supplementary Table S2
Characteristics of sources	Results 3.2; Table 1; Supplementary Table S3
Results of individual sources	Results 3.4; Table 2; Supplementary Table S3
Summary and limitations	Discussion 4.1–4.5
Conclusions	Section 5
Funding, conflicts, and data availability	Back matter

Supplementary Table S6. JBI critical appraisal of the included analytical cross-sectional studies

Study	Q1	Q2	Q3	Q4	Q5	Q6	Q7	Q8	Main appraisal considerations
Kılınç et al. 2014	Y	Y	Y	U	N	N	U	Y	Age range, dentition and recruitment setting were described. MLF classification was defined, but caries diagnostic criteria and examiner reliability were insufficiently reported. No confounder assessment or adjustment was performed.
Kınay Taran et al. 2023	Y	Y	Y	Y	U	N	U	Y	Eligibility, setting, Placek classification and WHO-based caries assessment were described. Potential confounders were not incorporated into an adjusted analysis, and reliability for caries assessment was not clearly reported.
Yilgör et al. 2025	Y	Y	Y	Y	Y	N	Y	Y	Inclusion and exclusion criteria were clearly stated. MLF and caries assessments were described, and intra- and inter-examiner reliability was evaluated. No multivariable adjustment was performed.
Kalaivanan et al. 2025	Y	Y	Y	Y	Y	N	Y	Y	Inclusion and exclusion criteria, ICDAS II assessment, examiner training and reproducibility assessment were reported. Potential determinants were recorded, but no adjusted analysis was performed. The sample-size discrepancy remains a reporting limitation.
Tokgöz Kaplan and Gökcek Taraç 2025	Y	Y	Y	U	N	N	U	Y	Eligibility criteria, setting, examiner experience and MLF classification were described. Caries was recorded as present or absent, but detailed diagnostic criteria and measurement reliability were not reported. No confounder adjustment was performed.
Kerins et al. 2026	Y	Y	Y	Y	Y	Y	U	Y	Eligibility criteria, setting, Kotlow classification and ICDAS assessment were described. Age and payer source were included in a multivariable model. Dentists were trained but not calibrated; diet, oral hygiene and fluoride exposure were not measured.

Checklist key. Q1: inclusion criteria clearly defined; Q2: study subjects and setting described in detail; Q3: exposure measured validly and reliably; Q4: objective standard criteria used for condition measurement; Q5: confounding factors identified; Q6: strategies to address confounding stated; Q7: outcomes measured validly and reliably; Q8: appropriate statistical analysis used. Y: Yes, N: No, U: Unclear or insufficiently reported. No overall numerical score or predefined cutoff was applied. The appraisal informed interpretation and was not used as an eligibility or exclusion criterion.
